# *SESN2* Could Be a Potential Marker for Diagnosis and Prognosis in Glioma

**DOI:** 10.3390/genes14030701

**Published:** 2023-03-12

**Authors:** Lingdan Xu, Zelin Liu, Huihui Wang, Jiyuan Lu, Jia Xu, Yucheng Meng, Ke Huang, Bin Liu

**Affiliations:** 1 Key Laboratory of Dental Maxillofacial Reconstruction and Biological Intelligence Manufacturing, School of Stomatology, Lanzhou University, Lanzhou 730030, China; 2 Key Laboratory of Preclinical Study for New Drugs of Gansu Province, School of Basic Medical Sciences, Lanzhou University, Lanzhou 730030, China

**Keywords:** glioma, *SESN2*, bioinformatics analysis, prognostic marker

## Abstract

(1) Background: Glioma is among the most common brain tumors, and is difficult to eradicate with current therapeutic strategies due to its highly invasive and aggressive characteristics. Sestrin2 (*SESN2*) is an autophagy inducer. The effect of *SESN2* on glioma is controversial and unclear. (2) Methods: We downloaded related RNA-seq data from the TCGA and GTEx databases. Bioinformatic analyses including differential gene expression analysis, KM survival curve analysis, univariate and multivariate Cox regression analyses, nomogram analysis, ROC curve analysis, gene function enrichment analysis, and immune cell infiltration analysis were conducted. In addition, data from the Human Protein Atlas (HPA) database were collected to validate *SESN2* expression in glioma. (3) Results: In comparison with normal tissue, expression of *SESN2* in glioma tissue was higher, and those with higher expressions had significantly lower overall survival rates. The results of univariate Cox regression analyses showed that *SESN2* can be a disadvantageous factor in poor glioma prognosis. Both nomograms and ROC curves confirmed these findings. Meanwhile, according to gene function analysis, *SESN2* may be involved in immune responses and the tumor microenvironment (TME). Based on the HPA database results, *SESN2* is localized in the cytosol and shows high expression in glioma. (4) Conclusions: The expression of *SESN2* in gliomas was positively relevant to a poorer prognosis, suggesting that *SESN2* could be used as a prognostic gene.

## 1. Introduction

Glioma is a common type of central nervous system (CNS) tumor [1], with an average survival period of only 12–15 months [2]. Due to its highly infiltrative and aggressive characteristics, it is difficult to eradicate using current treatment strategies including chemotherapy, radiotherapy, and surgery [3]. However, glioma is typically treated with surgical resection, supplemented by chemotherapy and radiotherapy [4]. Glioma is classified on the basis of the World Health Organization (WHO) classification system, from benign ependymomas to glioblastomas (GBMs) [5]. In the past decade, the development of new biomarkers for tumor diagnosis and treatment has presented a frontier of tumor-related research. Its diagnosis and treatment have become increasingly dependent on molecular biomarkers [6]. In order to improve the survival rate and prognosis of patients with glioma, a potential biomarker is urgently needed. As a diagnostic and prognostic indicator, it can help patients receive more efficacious treatments and increase their chances of survival.

Numerous studies have shown that the tumor microenvironment (TME) plays a considerable role in the progression of glioma [7], which constitutes tumor cells, immune cells, tumor-related stroma, and other components [8]. The TME, especially the tumor immune microenvironment (TIME), is one of the major factors affecting a patient’s prognosis [9]. The change in immune infiltration has a meaningful influence on the survival of glioma patients [10]. One study has shown that infiltrating immune cells can promote the development of tumors [11]. However, the prevailing view is that the immune system has the potential to both prevent and promote neoplastic growth [12]. Moreover, the interaction between immune cells and tumor cells is usually bidirectional. In detail, tumor cells can suppress the secretion of immune-related factors, and immune cells can kill tumors. Some genes have been found to be relevant to immune infiltration and affect tumor progression. Nevertheless, the relationship between *SESN2* and tumor immunity in glioma has not been explored.

Sestrins, with molecular weights between 52 and 57 kDa are known to belong to a highly conserved family [13]. Sestrins are expressed in mammalian cells in three isoforms: sestrin1 (*SESN1*), sestrin2 (*SESN2*), and sestrin3 (*SESN3*) [14]. *SESN2* was first described to be part of the hypoxia-induced gene 95 family (Hi95) [15] and is a powerful autophagy inducer able to activate the antioxidant system and maintain cell homeostasis [16]. The gene is present on chromosome 1, at Cytoband p35.3, and encodes 481 amino acids. The encoded protein of *SESN2* localizes to the cytosol. It has an antioxidant function [17], being involved in cell regeneration, protection, and survival [18,19]. *SESN2* negatively regulates the TORC1 signaling pathway by acting as an intracellular leucine sensor [18]. In most tumor-related studies, *SESN2* has anticancer effects [17]. It inhibits proliferation in hepatocytes [20], inhibits migration, and promotes autophagy in colorectal cancer cells [21,22]. There is a correlation between the low expression of *SESN2* and tumor progression and an unfavorable prognosis [17,19]. Despite the fact that *SESN2* generally antagonizes colon tumor growth, it can promote tumorigenesis in an iron-rich environment by suppressing cancer cell death associated with oxidative stress [23]. In contrast, *SESN2* promotes tumorigenesis and chemotherapy resistance in squamous cell carcinomas and melanoma [24]. A previous study showed ionizing radiation increased *SESN2* expression in human glioma U87 cells [25]. The effects of *SESN2* on human glioma cancer cells have not been fully examined, and its role in the prognosis of glioma remains unclear.

In the present study, we conducted comprehensive research on *SESN2* expression in glioma and evaluated its potential as a prognostic biomarker based on the TCGA database, and explore the role of related immune cells in glioma and possible molecular pathways. The findings of this study provide clinicians with additional information to help them better assess the long-term outcome prognosis of patients with gliomas.

## 2. Materials and Methods

### 2.1. Collecting and Processing Data

The Cancer Genome Atlas (TCGA) database was used to gather the gene expression profile data of 689 glioma samples and 5 peritumor tissues, while the Genotype-Tissue Expression (GTEx) provided 1,152 normal samples [26]. Concurrently, we incorporated glioma patient data from existing literature sources [27,28], leading to another 9 patient data points. TCGA provided RNA-seq data and related clinical information RNA-seq data. UCSC XENA was used to download the transcripts per million reads (TPM) format through Toil process standardization. To further compare tumor and normal mRNA levels, log2 fold change (log2FC) was calculated. Age, IDH status, 1p/19q status, WHO grade, histological type, and overall survival (OS) were included in the clinical data of glioma patients. We excluded unclear or incorrect samples to prevent unreliable results. It is important to understand how proteins function at the subcellular level. Protein expression and subcellular location of *SESN2* in glioma were verified through the Human Protein Atlas (HPA, https://www.proteinatlas.org/) database [29].

### 2.2. Survival and Statistical Analyses

Patients were divided into high expression and low expression groups in accordance with the median expression level of *SESN2*. The R software (version 3.6.3, R Core Team, Vienna, Austria) and the R package (survminer, version 0.4.9 and survival, version 3.2.10) were used to assess the correlation between *SESN2* expression level and overall survival via Kaplan–Meier (KM) survival analyses.

### 2.3. Univariate and Multivariate Cox Regression Analyses

We conducted univariate and multivariate Cox regression analyses to ascertain whether *SESN2* expression, race, age, gender, IDH status, 1p/19q status, WHO grade, and histological type were prognostic factors for the survival of glioma patients. We used the R package (survival, version 3.2.10) to process data. We calculated hazard ratios (HRs) and 95% confidence intervals (CIs), and the significance threshold was set at *p* < 0.05.

### 2.4. Construction of Nomograms, Calibration Plots, and ROC Curves 

Nomogram construction was performed in accordance with the nomogram guidelines [30]. The calibration of the nomogram was performed to visualize predicted probabilities’ deviations [31]. We used the R package (pROC, version 1.17.0.1 and ggplot2, version 3.3.3) to generate time-dependent receiver operating characteristic (ROC) curves for diagnostic analyses.

### 2.5. Analyses of SESN2-Related Gene Function Enrichment and Immune Cell Infiltration

Analyses of gene-gene correlations were carried out using the Spearman correlation function of the R package (stat, version 3.6.3). To screen and evaluate potential *SESN2*-related genes, the R package (org.Hs.eg.db, version 3.10.0 and clusterProfiler, version 3.14.3) were applied for Gene Ontology (GO) and Kyoto Encyclopedia of Genes and Genomes (KEGG) analysis. We used the R package (GSVA package, version 1.34.0) for immunocyte cell infiltration analyses.

## 3. Results

### 3.1. The Clinical Characteristics and SESN2 Expression of Glioma

The data of 698 primary tumors and 5 peritumor tissues were gathered from the TCGA database and relevant literature sources, while the data of 1152 normal samples were downloaded from the GTEx database. The clinical characteristics of glioma patients were collected, including WHO grade, age, IDH status, and histological type (Table 1). 

**Figure 1:**
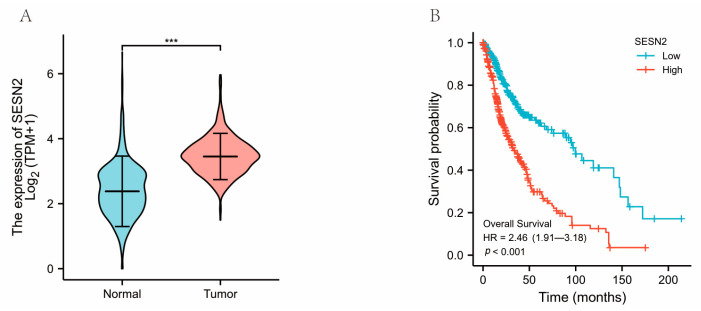
*SESN2*’s overall expression and survival analyses in gliomas. (**A**) *SESN2* was upregulated in glioma tumor tissues. (**B**) *SESN2* overexpression was involved in poor prognosis of glioma patients (*** *p* < 0.001).

In the present study, we compared the expression of *SESN2* between normal samples and glioma samples. The results showed that *SESN2* expression was significantly higher in glioma than in normal samples (*p* < 0.001) (Figure 1A). Then, KM survival analysis was conducted on glioma patients to determine the link between *SESN2* expression and overall survival. The overall survival of glioma patients with high *SESN2* expression was remarkably shorter based on KM survival curves (Figure 1B). Hence, a high expression level of *SESN2* is indicative of a worse prognosis outcome in gliomas.

### 3.2. Correlations of Clinical Characteristics with SESN2 Expression in Gliomas

**Figure 2:**
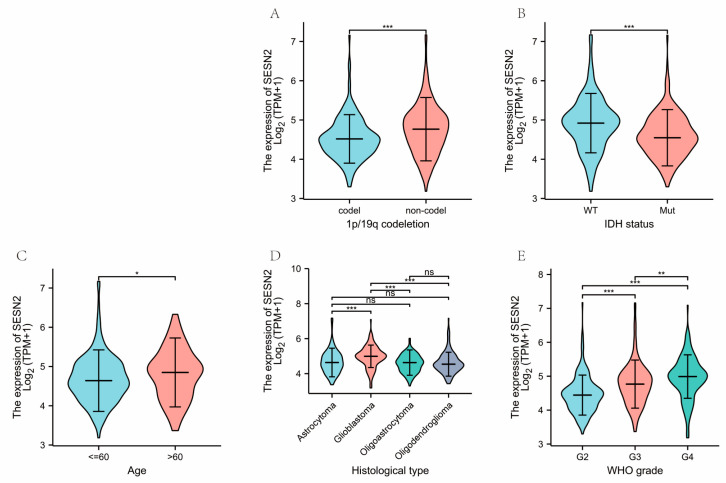
Associations between *SESN2* expression and clinical characteristics in glioma. (**A**) Correlations between *SESN2* expression and 1p/19q status. (**B**) Associations between *SESN2* expression and IDH status (WT: wild-type; Mut: mutation). (**C**) Associations between *SESN2* expression and age. (**D**) Associations between *SESN2* expression and histological type. (**E**) Associations between *SESN2* expression and WHO grade (ns, no significance; * *p* < 0.05; ** *p* < 0.01; *** *p* < 0.001).

Next, we explored the correlations between clinical characteristics and *SESN2* mRNA expressions in glioma. The results indicate the expression of *SESN2* was significantly different between the 1p/19q codeletion group and the non-codeletion group (*p* < 0.001) (Figure 2A). Secondly, *SESN2* was more expressed in the IDH mutation group than in the IDH wild-type group (*p* < 0.001) (Figure 2B). As for age, the expression of *SESN2* in patients above 60 years of age was slightly higher than in ages less than or equal to 60 years (*p* < 0.05) (Figure 2C). We also found varying *SESN2* expression levels in different histological types (Figure 2D). Of note, the level of *SESN2* expression was higher in high-grade gliomas (Figure 2E).

### 3.3. Associations between SESN2-Related Clinical Characteristics and Prognosis

**Figure 3:**
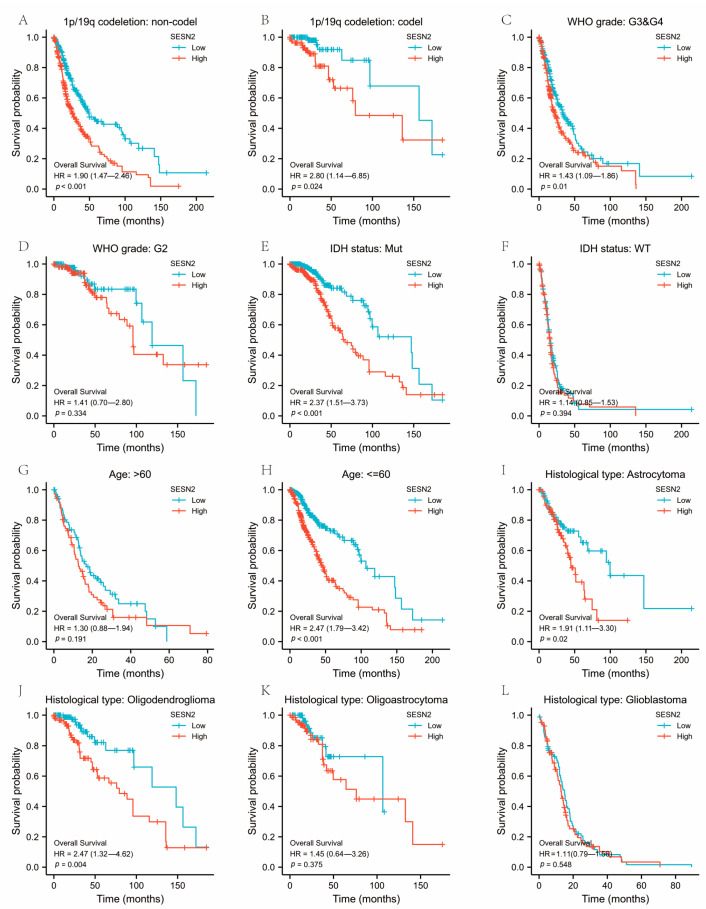
*SESN2* expression and survival analysis in glioma. (**A**,**B**) Subgroup survival analyses for correlations between *SESN2* expression and 1p/19q codeletion. (**C**,**D**) Subgroup survival analyses for correlations between *SESN2* expression and WHO grade. (**E**,**F**) Subgroup survival analyses for correlations between *SESN2* expression and IDH status. (**G**,**H**) Subgroup survival analyses for correlations between *SESN2* expression and age. (**I**–**L**) Subgroup survival analyses for correlations between *SESN2* expression and histological type.

We conducted a subgroup analysis to explore the prognosis of *SESN2* in different clinical contexts. In the 1p/19q non-codeletion status, the high expression of *SESN2* was markedly relevant to a poorer prognosis of glioma (Figure 3A), while in the 1p/19q codeletion status, they had a relatively small correlation (Figure 3B). High expression of *SESN2* was associated with a worse prognosis in grades WHO III and IV, while in grade II, no correlation was observed (Figure 3C,D). Meanwhile, high expression of *SESN2* was significantly relevant to a worse prognosis in mutant IDH status and older patients (Figure 3E–H). As for the histological types, high expression of *SESN2* was relevant to a poorer prognosis in astrocytoma and oligodendroglioma (Figure 3I,J). We did not find a remarkable relationship between *SESN2* expression and the prognosis of oligoastrocytoma and glioblastoma (Figure 3K,L).

### 3.4. Diagnostic Value of SESN2 in Glioma

**Figure 4:**
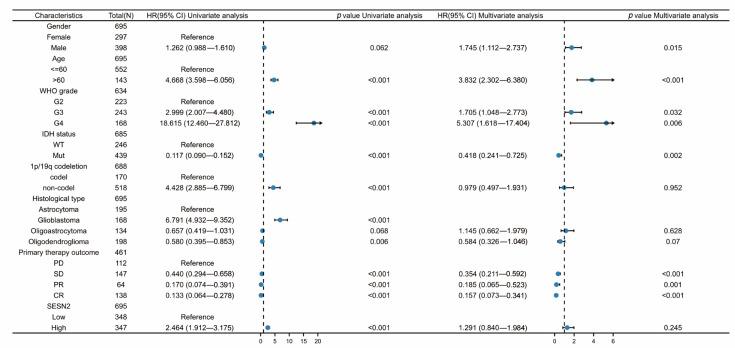
Univariate analysis and multivariate analysis risk score of *SESN2* expression and relevant key clinical features. An HR above 1 indicates an increased risk, while an HR below 1 indicates a protective effect (PD: progressive disease; SD: stable disease; PR: partial response; CR: complete response).

**Figure 5:**
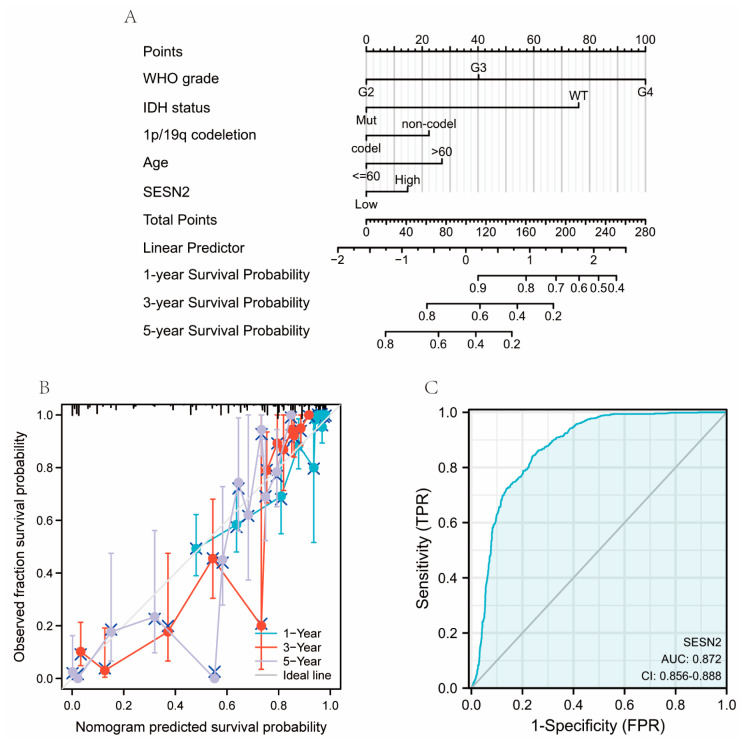
Diagnostic value of *SESN2* in glioma. (**A**) The nomogram was developed by integrating the *SESN2* expression with key clinical characteristics. (**B**) The calibration plot of the nomogram for predicting overall survival at 1-year, 3-years, and 5-years. (**C**) The diagnostic value of *SESN2* (AUC, 0.872; 95%CI, 0.856-0.888).

We used univariate and multivariate Cox regression analysis to identify risk factors. Based on the univariate Cox regression analysis, high *SESN2* expression was associated with a poorer outcome in gliomas (HR = 2.464, 95% confidence interval (CI), 1.912–3.175; *p* < 0.001). However, the results of the multivariate analysis showed no statistical significance (*p* > 0.05) (Figure 4). Hence, *SESN2* is a prognostic gene of glioma. Additionally, there were risk factors related to age, IDH status, 1p/19q status, WHO grade, histological type, and primary therapy outcome. Using these prognosis factors, we constructed nomograms predicting 1, 3, and 5-year survival rates (Figure 5A). The nomogram-predicted survival probability calibration plots were highly close to the ideal (Figure 5B). The ROC curve was applied to evaluate the diagnostic performance of SESN2. The area under the curve (AUC) was 0.872 (95% confidence interval (CI), 0.856–0.888; *p* < 0.001), indicating good discrimination between patients with and without the target condition (Figure 5C). The sensitivity was 84%, and the specificity was 76.1% when the cut-off point was 2.946. *SESN2* was shown to have an excellent diagnostic effect on glioma.

### 3.5. Co-Expression of Genes Responsible for SESN2 and Predicted Gene Functions

**Figure 6:**
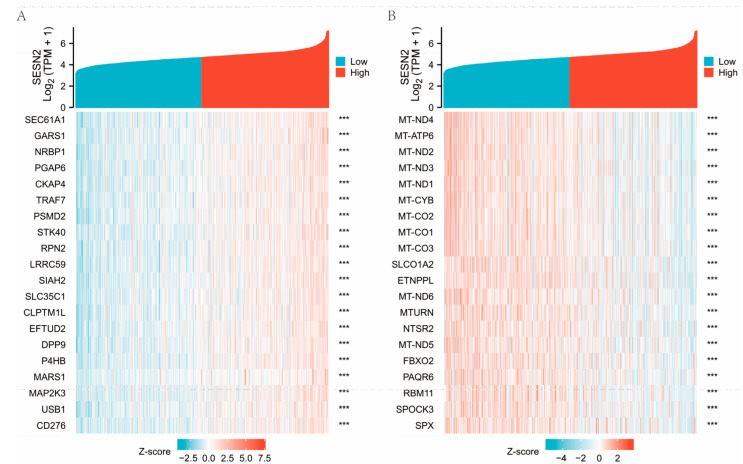
Heat maps illustrating patterns of gene co-expression. (**A**) Top 20 positive correlation genes. (**B**) Top 20 negative correlation genes (*** *p* < 0.001).

**Figure 7:**
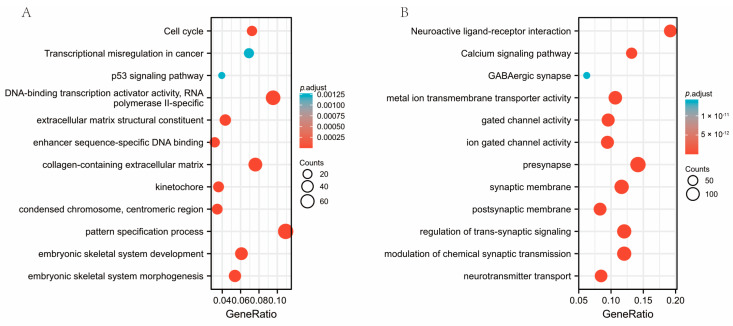
Bubble plot of GO/KEGG enrichment analysis. (**A**) The up-regulated gene of *SESN2*. (**B**) The down-regulated gene of *SESN2*.

The top 40 relevant genes identified via gene correlations are presented (Figure 6A,B). GO and KEGG enrichment analysis was conducted in order to investigate the possible biological functions of *SESN2*. Activating functions related to *SESN2* were found in the results, such as cell cycle, DNA-binding transcription activator activity, RNA polymerase II-specific, collagen-containing extracellular matrix, and the pattern specification process (Figure 7A). Meanwhile, *SESN2* downregulates the expression of active genes relevant to neuroactive ligand-receptor interaction, metal ion transmembrane transporter activity, synaptic membrane, regulation of trans-synaptic signaling, calcium signaling pathway, and modulation of chemical synaptic transmission (Figure 7B).

### 3.6. The Association between SESN2 and Immune Cell Infiltration in Glioma

**Figure 8:**
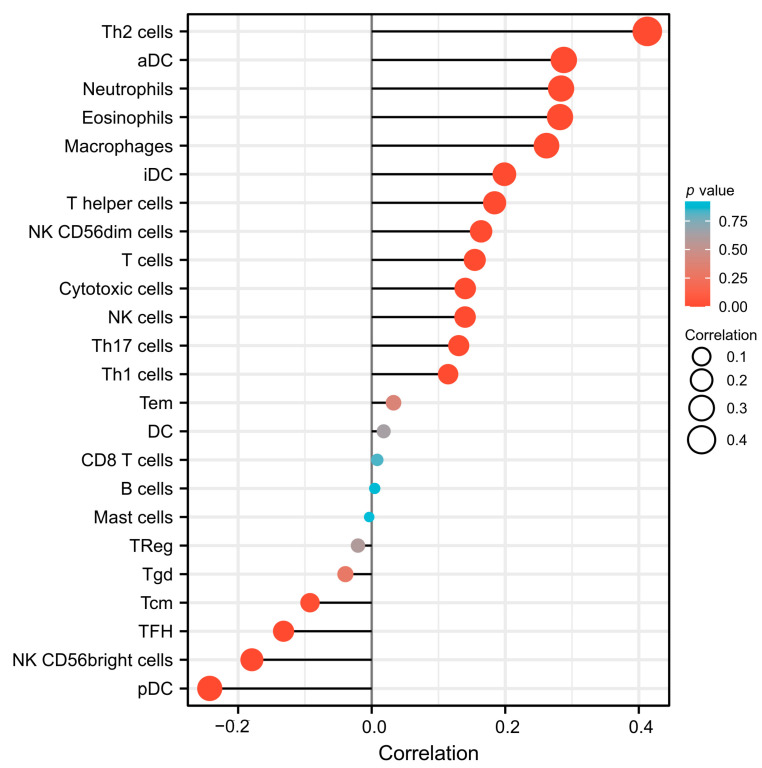
The association of *SESN2* with 24 types of immune cells in glioma according to TCGA data.

Following the above analyses, further analysis of *SESN2* expression and immune cell infiltration was conducted. There are 24 types of immune cells represented by the data downloaded from the TCGA database. There was an obvious positive correlation between *SESN2* expression and Type 2 helper T (Th2) cells, aDC, Neutrophils, Eosinophils, Macrophages, interdigitating dendritic cell (iDC), T helper cells, and NK CD56 dim cells (Figure 8). At the same time, there is a significant negative correlation with pDC, NK CD56 bright cells, and follicular T helper (TFH) cells (Figure 8).

### 3.7. Subcellular Location and Protein Expression

**Figure 9:**
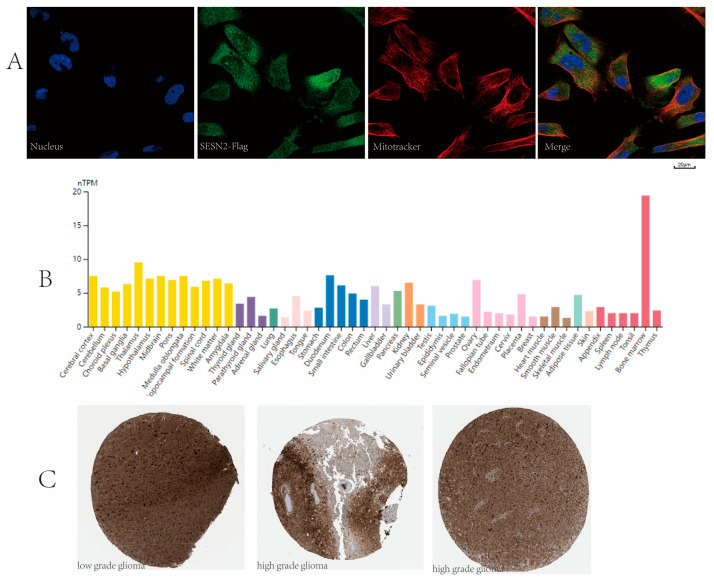
(**A**) Immunofluorescent staining of the U251-MG cell line showed localization to the cytosol. (**B**) *SESN2* expression in different normal human tissues. Moderate expression of *SESN2* was detected in normal cerebral cortex tissues. (**C**) Immunohistochemical staining images of *SESN2* in glioma. All the pictures were downloaded from the HPA database. Antibody HPA018191 was used for staining.

*SESN2* is localized to the cytosol according to the HPA database (Figure 9A). As shown in the expression profile of *SESN2* from HPA, moderate expression of *SESN2* was detected in normal cerebral cortex tissues (Figure 9B). Meanwhile, the immunohistochemical results revealed that strong *SESN2* expression was also recorded in gliomas (Figure 9C).

## 4. Discussion

The prognosis of glioma, particularly high-grade glioma, is poor due to its high invasiveness. Gliomas account for 81% of central nervous system malignancies, with 5 to 10 cases per 100,000 people [32]. In low-grade gliomas, the age-standardized 10-year survival rate was 47%, and the median survival was 11.6 years [33,34]. For high-grade gliomas, the median overall survival was about 3 years for anaplastic astrocytoma and 15 months for glioblastoma multiforme [35]. To improve the cure rate of the disease compared with the patient survival rate, a potential biomarker, and therapeutic target are urgently needed as both diagnostic and prognostic indicators. To explore more possible biomarkers for glioma, we performed a search of the literature and found the IDH1 G105G SNP. However, it was not found to have any prognostic impact on IDH wild-type Glioblastoma [36].

Previous studies indicated that *SESN2* could alleviate oxidative stress via nuclear factor erythroid 2-related factor 2 (Nrf2), and thus favor cancer cell survival [37]. According to this study, *SESN2* expression was observably increased in gliomas, while its high expression was markedly relevant to a poor prognosis. The expression of *SESN2* in gliomas with varying clinical features was analyzed. In gliomas, IDH mutations distinguish WHO grade 1 glioma from glioma proliferation [35], indicating a relatively good prognosis [38,39]. *SESN2* was more highly expressed in IDH mutation than in IDH wild-type (*p* < 0.001). Chromosome 1p/19q co-deletion (codel) is a key variant in oligodendrogliomas [35], which indicates a relatively favorable prognosis [40]. The expression of *SESN2* had a significant difference between 1p/19q codeletion and non-codeletion (*p* < 0.001). In all, a high expression of *SESN2* is markedly relevant to IDH status, WHO grade, 1p/19q status, and histological type. Further, we performed the subgroup analysis of the effect of *SESN2* on prognosis. The results indicated that high expression of *SESN2* was significantly relevant to a poor prognosis in IDH mutations, 1p/19q non-co-deleted glioma, and glioma patients above 60 years of age. Furthermore, the results of univariate and multivariate Cox regression analyses, nomograms, calibration plots, and ROC curves in combination, suggest SESN2 could be used as a predictor of a poor prognosis. In general, univariate analysis and multivariate analysis complement each other. In multivariate Cox regression analysis, the inclusion of more variables complicates interpretation. In this paper, the results of the univariate analysis showed statistical significance, but the results of the multivariate analysis showed no statistical significance. In that case, we believe that *SESN2* is a disadvantageous factor rather than an independent prognostic factor.

Sestrins are believed to be an essential component of antioxidant defense [41]. In all, the role of *SESN2* in excessive oxidative stress, hypoxia, DNA damage, amyloid-induced, and mitochondrial dysfunction neurotoxicity has been investigated [42,43,44,45]. The role of *SESN2* in glioma is still unclear. Therefore, we investigated the gene functions relevant to *SESN2* in glioma. The outcomes suggest that potential biological functions may include neuroactive ligand-receptor interaction, synaptic membrane, synaptic signaling regulation, calcium signaling pathways, cell cycle, DNA-binding transcription activator activity, RNA polymerase II-specific, collagen-containing extracellular matrix, and pattern specification process, and presynaptic signaling regulation (Figure 7). Studies have found glioma cells and TME cells communicate through the interaction of neuroactive ligand-receptors [46,47]. Metabotropic glutamate receptor 1 (GRM1), 5-Hydroxytryptamine (serotonin) receptor 7 (HTR7), and 5-Hydroxytryptamine (5-HT) receptor 2A (HTR2A) may play a role in glioma by participating in the interaction pathway of neuroactive ligand receptor interaction pathway [47,48]. HTR2A has also been confirmed to be associated with glioma grade through neuroactive ligand receptor interaction [49]. Neurons and glioma cells can communicate directly through chemical synapses. Furthermore, glutamate synaptic input into glioma cells promotes the development of glioma [50]. Our study also found that *SESN2* can down-regulate synapse-related pathways to affect glioma. Astrocytoma conditioned medium can promote the elimination of cell synapses and have a negative impact on the stability of synapses [51]. Synaptic vesicular protein 2A can be used as a predictor of the response of glioma patients to certain drugs [52]. In addition, GRM1 and adenylate cyclase 2 (ADCY2) may contribute to glioma development through the calcium signaling pathway [46,47]. The cell cycle, which is regulated by HOXD-AS2, can promote the development of glioma [53]. Meanwhile, Calmodulin Binding Transcription Activator 1 (CAMTA1) plays a vital role in the human nervous system and affects the prognosis of glioma. Our study found that the potential biological function of *SESN2* can up-regulate the cell cycle and DNA-binding transcriptional activator activity related pathways, thus affecting the onset and progression of glioma. As for the collagen-containing extracellular matrix, Zhou’s study showed that Mitochondrial Ribosomal Protein S17 (MRPS17) can promote tumor cell metastasis through this pathway in gastric cancer [54]. Additionally, the excessive collagen-containing extracellular matrix is a hallmark of fibrosis [55]. 

The TIME contains a large number of tumor immune cell infiltration, which are significantly related to tumor progression [56]. The level of infiltration of immune cells varies in different glioma classifications and stages [10]. In TIME, immune cells have abnormal components and functions that contribute to immune escape, drug resistance, and metastasis, which is the key reason why tumors are difficult to cure. Creating an immunosuppressive environment can help glioma cells escape immune surveillance and immune attack [57], which is a characteristic of glioma TIME. In this study, we examined how *SESN2* expression relates to immune cell infiltration. The results show that the expression of *SESN2* was positively correlated with Th2 cells, aDC, neutrophils, eosinophils, macrophages, iDCs and T helper cells, and NK CD56 dim cells. Meanwhile, the expression of *SESN2* was negatively relevant to pDC, NK CD56 bright cells, and TFH. Th2 cells belong to CD4 + T cells [58]. Previous studies have shown that in glioma, conversion from Th2 to Th1 helps prevent the growth of glioma [59]. The adaptive response of Th2 will affect the response of Th1, so the balance of Th1/Th2 is very important [60]. In gastric cancer, similar to Th2 cells, macrophages can also secrete interleukin-10 (IL-10) and participate in immunosuppression [61]. DCs are specialized antigen-presenting cells that are involved in antitumor immunity [62]. NK cells were divided into two subtypes in accordance with the density of CD56. The CD56 bright phenotype can differentiate into the CD56 dim phenotype [63]. In glioma, the expression of *SESN2* showed a positive correlation with NK CD56 dim phenotype and a negative correlation with the CD56 bright phenotype in the present study. We speculate that the reason for this result may be that the high expression of *SESN2* promotes this transformation. Our study also found an increase in neutrophil and eosinophil infiltration. Neutrophils account for 50–70% of all white blood cells and are an important defense line of human immunity. Meanwhile, eosinophils account for 0.5–5%, and infiltration is observed in various tumors [64,65]. The degree of eosinophil infiltration in tumor is closely related to the number of eosinophils in the blood [66]. In general, the exact relationship between *SESN2* and the glioma immune microenvironment necessitates further research to explore.

Taken together, our study clarified the relationship between *SESN2*’s high expression and the clinical features of glioma. *SESN2* can be considered a potential marker for the diagnosis and prognosis of glioma. We discussed the potential biological function of *SESN2* in glioma and its correlation with immune cell infiltration. However, as with any research design, our approach also has its limitations. Firstly, our conclusions were not confirmed by in vitro or in vivo experiments. Moreover, researchers have not deeply explored the causes and mechanisms of high *SESN2* expression in glioma patients. There is no clear explanation for the differential expression in gliomas with varying clinical features. Finally, there is no systematic and in-depth study on the relevant mechanism between the high expression of *SESN2* and the infiltration of immune cells. In order to understand the molecular mechanism underlying *SESN2* in gliomas, further studies are needed.

## 5. Conclusions

The expression of *SESN2* is higher compared with normal tissue in the present study. Moreover, the high *SESN2* expression was significantly correlated with a poor outcome in glioma, which suggests that *SESN2* could be a potential marker for diagnosis and prognosis. Further experimental assays are necessary to validate the conclusion. We infer these results may be mechanistically linked to alterations in the immune microenvironment.

## Figures and Tables

**Table 1:** The clinical characteristics of glioma patients.

Characteristic	Levels	Overall
WHO grade, n (%)	G2	224 (35.3%)
	G3	243 (38.3%)
	G4	168 (26.5%)
IDH status, n (%)	WT	246 (35.9%)
	Mut	440 (64.1%)
Histological type, n (%)	Astrocytoma	195 (28%)
	Glioblastoma	168 (24.1%)
	Oligoastrocytoma	134 (19.3%)
	Oligodendroglioma	199 (28.6%)
Age, n (%)	≤60	553 (79.5%)
	>60	143 (20.5%)

## Data Availability

Not applicable.
